# Risk-adjusted chemoradiation according to human papilloma viral status for anal cancer: a pilot study

**DOI:** 10.3389/fonc.2023.1183854

**Published:** 2023-06-29

**Authors:** William Chu, Amandeep Taggar, Yee Ung, Kelvin K. W. Chan, Craig C. Earle, Aliaksandr Karotki, Mark Pasetka, Joe Presutti, John Wong, Liying Zhang, C. Shun Wong

**Affiliations:** ^1^Department of Radiation Oncology, Sunnybrook Health Sciences Centre, University of Toronto, Toronto, ON, Canada; ^2^Department of Medicine, Sunnybrook Health Sciences Centre, University of Toronto, Toronto, ON, Canada; ^3^Institute for Clinical Evaluative Sciences, Dalla Lana School of Public Health, University of Toronto, Toronto, ON, Canada; ^4^Department of Medical Physics, Sunnybrook Health Sciences Centre, Toronto, ON, Canada; ^5^Department of Pharmacy, Sunnybrook Health Sciences Centre, Toronto, ON, Canada; ^6^Department of Radiation Therapy, Sunnybrook Health Sciences Centre, Toronto, ON, Canada; ^7^Department of Laboratory Medicine and Molecular Diagnostics, Sunnybrook Health Sciences Centre, University of Toronto, Toronto, ON, Canada; ^8^Department of Radiation Oncology, Sunnybrook Health Sciences Centre, Toronto, ON, Canada

**Keywords:** anal cancer, human papilloma virus, chemoradiation, risk-adjusted therapy, IMRT

## Abstract

**Background and purpose:**

HPV-associated or positive (HPV+) anal cancer patients may have better outcome compared to those with HPV negative (HPV−) disease. We report a planned interim analysis of a prospective registry study that tailors chemoradiation (CRT) for anal cancer according to HPV status.

**Materials and methods:**

HPV+ patients received de-escalated radiation doses of 45, 50.4 and 55.8 Gy, while HPV− received 50.4, 55.8 and 63 Gy for T1, T2 and T3/T4 disease respectively. Chemotherapy consisted of a single dose of mitomycin-C and oral capecitabine on days of RT. All patients were planned by VMAT following CT, PET/CT and MR simulation. This cohort (n = 24) had a minimum 24-month follow-up. Disease free survival (DFS) and local failure rates (LFR) were compared with 180 patients managed by standard CRT (2 cycles of mitomycin-C and 5-fluorouracil, radiation doses 50.4-63 Gy based on T-category) from 2011-2018. Propensity score comparison was performed using a retrospective to prospective 2 to 1 match based on tumor size and N-category.

**Results:**

In the HPV+ cohort (n = 20), there were 2 local failures. Two of 4 HPV− patients failed locally. The 30-month DFS and LFR were 79% and 17% respectively. Similar DFS and LFR were observed in the retrospective (80% and 15% respectively) and matched patients (76% and 16% respectively). No grade ≥3 neutropenia and febrile neutropenia were observed in the registry cohort whereas 19% and 14% respectively were seen in the retrospective patients.

**Conclusion:**

De-escalation of CRT for HPV+ anal cancer may result in decreased acute toxicities and similar cancer outcomes compared to standard CRT.

## Introduction

Epidermoid anal carcinomas represent a unique tumor type in that radiation (RT) with concurrent mitomycin C (MMC) and 5-fluorouracil (5FU), modified after the regimen originally reported by Nigro (1) has replaced abdominoperineal resection (APR) as standard treatment (2). The superiority of concurrent MMC-5FU with RT over RT alone and other concurrent CRT regimens were subsequently confirmed in randomized trials (3–7). In addition, induction chemotherapy or maintenance chemotherapy does not confer any improvement in outcome compared to RT with concurrent MMC-5FU (6, 8, 9). Excellent tumor control can be achieved in early-stage cancer with CRT, however, local failure remains a challenge in patients with locally advanced anal cancer (2). At present, there is no evidence to suggest that dose escalation beyond 55-60 Gy is associated with improved outcome in locally advanced cancers (8, 10). Many patients experience acute and late toxicities following CRT (2).

Since 2011, at our institution anal cancer has been managed by a prospectively designed protocol of CRT consisting of doses-escalated intensity modulated radiotherapy (IMRT) according to increasing tumor stages without a planned RT break (11). Unfortunately despite IMRT, significant acute skin/perineal reactions and grade ≥3 hematologic toxicities and febrile neutropenia were observed. Thus, further efforts were deemed necessary to optimize available treatment protocols not only to improve tumor control, but also to reduce acute and late treatment morbidities.

The majority of anal cancers are associated with high-risk human papilloma virus (HPV). Emerging evidence suggests that HPV-associated or positive (HPV+) cancers including anal cancers have increased radiosensitivity and improved prognosis (12–14). In 2019, we instituted a prospective anal cancer registry study with the CRT protocol tailored according to HPV status in addition to tumor stages. The goal was to determine if toxicities could be reduced without negative impact on tumor control following a de-escalation CRT protocol in patients with HPV+ cancer. Here we report results of a planned interim analysis after the initial cohort of 24 consecutive participants had a minimum follow up of 24 months. Patient outcomes were compared with our retrospective cohort of 180 patients treated since the introduction of IMRT as a standard in 2011 (11).

## Materials and methods

This is a single institution registry study approved by the institutional Research Ethics Board (REB). All participants signed a REB approved informed consent (REB Project ID: 133-2019). The primary endpoint was 2-year local failure rate (LFR). The secondary endpoints were acute and late toxicities, disease-free survival (DFS), colostomy-free survival (CFS) and overall survival (OS).

Eligibility criteria included: 1) histologically confirmed diagnosis of epidermoid/squamous cell carcinoma of anal canal or perianal skin (defined as cancer from the anatomic anal margin); 2) known p16 status by immunohistochemistry and HPV status by PCR; 3) any T and N category; 4) eligible for definitive RT with or without concurrent MMC) and capecitabine. Patients <18 years of age or with history of previous pelvic RT, or with life expectancy <6 months were ineligible. All patients were staged by CT-chest/abdomen/pelvis, MRI-pelvis and PET/CT, and reviewed at the institutional lower gastrointestinal (GI) multidisciplinary case conference prior to CRT.

Detection of HPV was performed in DNA extracted from formalin-fixed paraffin embedded samples using the Cobas-4800 HPV test system (Roche, Rotkreuz, Switzerland). Tumors were recorded as (HPV+) or negative (HPV−) for HPV type 16, 18 or other high-risk types as the system cannot distinguish between high-risk types of 31, 33, 35, 39, 45, 51, 52, 56, 58, 59, 66 or 68 from one another. The detection limits for HPV type 16 and 18 are 300 and 600 copies/mL, respectively. Detection limits for the other 12 high risk types vary from 100 to 7200 copies/mL Biopsies were reviewed by our institutional GI pathologists. Status for p16 was based on immunohistochemistry as per the institutional reporting pathologist.

The RT protocol according to T and N category and HPV/p16 status is outlined in **Table 1**. All patients were simulated by a planning CT followed immediately by a planning MR. A planning PET/CT was performed within 2-3 days of the planning CT. Contouring of GTV/CTV/PTV and OARs was as per departmental protocol modified from the RTOG contouring guidelines with standardized contouring and planning nomenclature (15). Concurrent chemotherapy consisted of MMC (12 mg/m^2^, maximum 20 mg) on day 1 of RT, and capecitabine (825 mg/m^2^ bid) daily on days of RT only. RT contouring and treatment plans were reviewed at quality assurance rounds as per departmental policy. All patients were treated using volumetric modulated arc therapy (VMAT) using shrinking fields sequential boost technique. There were no planned RT breaks. There were no T1 perianal (margin) cancers in both cohorts as they were managed by local excision as per institutional treatment policy.

**Table 1:** Summary of dose fractionation schedules* according to tumor stage and human papilloma virus (HPV) status in the prospective registry.

HPV+						
	Phase 1		Phase 2		Total	
	Dose (Gy)	Fractions	Dose (Gy)	Fractions	Dose (Gy)	Fractions
T0	30.6	17	0	0	30.6	17
T1, <2 cm	30.6	17	14.4	8	45	25
T2, 2-5 cm	30.6	17	19.8	11	50.4	28
T3, >5 cm	30.6	17	25.2	14	55.8	31
T4	30.6	17	25.2	14	55.8	31
N0	30.6	17	0	0	30.6	17
N+, <2 cm	30.6	17	14.4	8	45	25
N+, 2-5 cm	30.6	17	19.8	11	50.4	28
N+, >5 cm	30.6	17	25.2	14	55.8	31
HPV− or status unknown						
	Phase 1		Phase 2		Total	
	Dose (Gy)	Fractions	Dose (Gy)	Fractions	Dose (Gy)	Fractions
T0	36	20	0	0	36	20
T1, <2 cm	36	20	14.4	8	50.4	28
T2, 2-5 cm	36	20	19.8	11	55.8	31
T3, >5 cm	36	20	27	15	63	35
T4	36	20	27	15	63	35
N0	36	20	0	0	36	20
N+, <2 cm	36	20	14.4	8	50.4	28
N+, 2-5 cm	36	20	19.8	11	55.8	31
N+, >5 cm	36	20	27	15	63	35

*Participants may receive multiple phases of RT based in size of primary and regional nodes.

Acute toxicities were graded according to CTCAE version 4.02 weekly during CRT. Grade ≥3 acute toxicities were recorded weekly until resolution. Late toxicities were recorded at follow up clinics according to the RTOG late toxicity scores.

Patients were followed 1-month post-CRT, then every 3 months for 2 years, every 6 months for 3 years, and yearly for another 5 years thereafter. Follow-up included digital rectal exam, CT and MRI at 6 months from start of CRT based on results of ACT II trial (2). For patients with a complete response, subsequent follow-up CT and MRI were performed every 6 months thereafter for 2 years, and yearly at year 3-5. For patients with suspicion of residual or recurrent disease on digital exam or MRI were referred for endoscopic evaluation and biopsy.

Twenty-five patients were referred to our institution during the accrual period for treatment of anal cancer. One patient (T2N0) declined to participate in the study and was excluded. Thus 24 consecutive participants, all of whom had a minimum of 24 months of follow up from date of CRT were included in this analysis. A single participant had M1 disease due to common iliac nodal metastasis that could be treated definitively within the RT volume and was included in the study.

Outcomes were compared with a retrospective cohort of 180 patients with anal cancer treated between 2011 and 2019 at our institution (**Supplementary Table 1**). These patients were managed by a prospectively designed CRT protocol since IMRT became standard in 2011. Patients received total RT doses escalated according to increasing tumor stages, namely total doses of 50.4, 55.8 and 63 Gy for T1, T2 and T3/T4 disease respectively, and 36 Gy for elective nodal RT. Involved nodes were given the same dose based on T category of the disease. There was no planned treatment break. Concurrent chemotherapy consisted of two cycles of MMC (10 mg/m^2^, maximum 20 mg) and 5-fluorouracil (5FU, 1000 mg/m^2^/day x 4 days) given on week 1 and 5. HPV typing and p16 immunohistochemistry were not routinely done. Staging PET/CT was also not routinely performed. Planning was performed by CT only.

### Statistical analysis

Patient and treatment characteristics were summarized as mean, standard deviation (SD), median, interquartiles, and range for continuous variables, count and proportions for categorical variables. Kaplan Meier methods were used to describe overall survival (OS), disease free survival (DFS) and colostomy free survival (CFS). Comparison of these endpoints in the registry cohort and retrospective cohort of patients was performed by the log-rank test. Cumulative incidence of local failure rates (LFR) was estimated and compared between the registry study and retrospective study using Gray’s test. A propensity matched pair comparison (16) was performed by a 2 to 1 match of patients from the retrospective cohort to the prospective cohort using tumor size and N-stage. Propensity score weighting was based on tumor size (<2, 2-<4, 4-<6, ≥6 cm) and N stage (0, N+). A p-value <0.05 was considered statistically significant. Wilcoxon rank-sum test or Fisher exact test was used for continuous or categorical variables on evaluating the differences between these two groups. All analyses were conducted using Statistical Analysis Software (SAS version 9.4, Cary, NC) or R package (v4.2.1).

## Results

The median age of the 24 participants was 67 (49-86, median 65). **Table 2** outlines the clinical and tumor characteristics of the 24 patients. Twenty of 24 patients had HPV+ cancer. Sixteen were HPV16+, one was HPV18+, and the remaining 3 had other high-risk HPV types. All 20 HPV+ lesions demonstrated immunoreactivity for p16. None of the 4 HPV− lesions were p16+.

**Table 2:** Patient and tumor characteristics of registry participants (n = 24).

Patient/tumor characteristic	Number of patients (%)	
Sex		
Male	4	(17%)
Female	20	(83%)
ECOG performance status		
0-1	21	(88%)
2	2	(8%)
3	1	(4%)
Histological grade		
1	1	(4%)
2	6	(25%)
3	2	(8%)
Unknown	15	(63%)
p16 immunoreactivity		
No	4	(17%)
Yes	20	(83%)
HPV status		
Negative	4	(17%)
Type 16	16	(67%)
Type 18	1	(4%)
Other high-risk types*	3	(12%)
Site		
Anal canal	21	(88%)
Perianal (anal margin)	3	(12%)
HIV positive		
No	23	(96%)
Yes	1	(4%)
T-category		
1	1	(4%)
2	14	(58%)
3	5	(21%)
4	4	(17%)
N-category		
0	12	(50%)
1a	5	(21%)
1b	2	(8%)
1c	5	(21%)
M-category		
0	23	(96%)
1**	1	(4%)

*HPV type 31, 33, 35, 39, 45, 51, 52, 56, 58, 59, 66 or 68.

**common iliac nodal metastasis.

In the HPV+ cohort (n = 20), there were 1 T1 (1.6 cm), 11 T2 (2.1-4.8 cm), 5 T3 (5.1-5.8 cm) and 3 T4 (4-8.5 cm) lesions. All 3 patients with T4 disease demonstrated invasion of the vagina. Eight patients (N1a, 4; N1b, 1; N1c, 3) presented with nodal metastases. Among the 4 patients with HPV− disease, there were 3 T2 (3.2-4 cm) and 1 T4 (9.1 cm) lesions, and 2 had nodal disease (1 N1a and 1 N1c). The median greatest dimension of the primary based on MRI was 4 cm (1.6-9.1 cm, mean 4.5 cm). The median greatest dimension of regional nodal metastasis was 1.5 cm (0.6-5.3 cm, mean 2.0 cm).

A single patient (HPV−,T4N0) underwent a colostomy prior to CRT. Three patients did not receive concurrent chemotherapy and one had capecitabine only due to medical comorbidities or patient refusal. One HPV− patient (T2N1c) required a 9 day treatment gap. All 24 patients completed RT as per protocol.

All 24 patients experienced at least grade 2 acute toxicities. **Table 3** outlines the acute toxicities. Grade 3 toxicities (skin reactions, pain and anemia) were observed in 5 of 20 HPV+ patients and 3 of 4 HPV− patients. One patient (HPV−, 63 Gy) developed a rectal perforation associated with an ischemic rectosigmoid at one year requiring multivisceral resection. No other grade ≥3 late toxicities were otherwise observed.

**Table 3:** Acute toxicity grades in registry participants (n = 24) managed by chemoradiation according to tumor stage and HPV status.

Toxicity grade	Number of patients (%)	
Fatigue		
0	1	(4%)
1	10	(42%)
2	13	(54%)
Anorexia		
0	11	(46%)
1	6	(25%)
2	7	(29%)
Nausea		
0	15	(63%)
1	7	(29%)
2	2	(8%)
Vomiting		
0	21	(88%)
1	2	(8%)
2	1	(4%)
Diarrhea		
0	12	(50%)
1	9	(37%)
2	3	(13%)
Dehydration		
0	20	(83%)
1	1	(4%)
2	1	(4%)
3	2	(8%)
Stomatitis		
0	21	(88%)
1	2	(8%)
3	1	(4%)
Proctitis		
0	6	(25%)
1	11	(46%)
2	7	(29%)
Cystitis		
0	19	(79%)
2	5	(21%)
Pain		
0	1	(4%)
1	5	(21%)
2	15	(63%)
3	3	(12%)
Skin		
1	3	(13%)
2	14	(58%)
3	7	(29%)
Anemia		
0	22	(97%)
3	2	(8%)
Neutropenia		
0	19	(79%)
2	5	(21%)
Thrombocytopenia		
0	24	(100%)
Febrile neutropenia		
0	24	(100%)
Other acute toxicity grade		
0	21	(88%)
2	1	(4%)
3	2	(8%)
Any acute toxicity grade ≥3		
No	16	(67%)
Yes	8	(33%)

**Figure 1:**
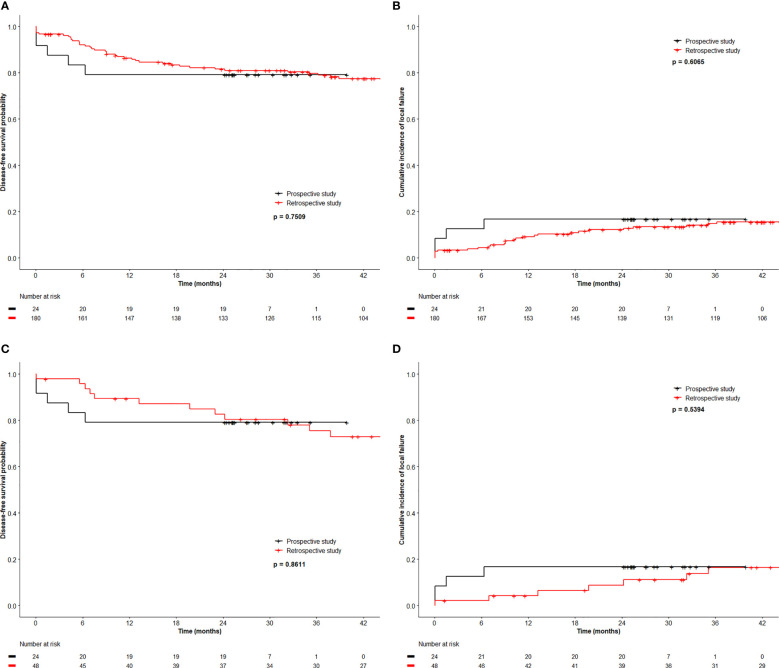
Disease free survival **(A)** and local relapse free rate **(B)** of registry patients and retrospective patients, and disease free survival **(C)** and local relapse free rate **(D)** of registry patients and 2 to 1 matched retrospective patients.

All patients underwent restaging MRI at a median of 170 days (103-204 days) from start date of RT. Among the 20 HPV+ patients, there were 2 (T4N1c and T2N0) local failures. They represented the only 2 patients with persistent or residual local disease on restaging MRI. One (T2N0) underwent a salvage abdominoperineal resection and remained free of recurrence 2 years after salvage surgery. Of 4 patients with HPV− cancer, one (T2N1b) had persistent local disease and one (T2N1c) failed in the iliac nodes without relapse at the primary site. Both died of disease. The 30-month OS, DFS, CFS and LFR were 91%, 79%, 92% and 17% respectively (See **Figures 1A, B** for DFS and LFR).

We compared the outcome of the prospective registry patients (n=24) with our retrospective cohort of 180 patients managed by CRT with a RT dose schedule currently used for HPV− patients. A comparison of the clinical characteristics revealed no significant difference between both cohorts (**Table 4**). The median size of the primary was 4.0 cm (0.6 – 11 cm, mean 4.3 cm) in the retrospective cohort (p=0.6 compared with the registry patients). In the retrospective cohort, 43 (24%) required a treatment break (6-33 days) principally due to acute toxicities. The overall treatment time (48.6 ± 10.4 days) in the retrospective cohort was significantly longer compared to the registry participants (42.2 ± 5.0 days, p < 0.001). The local failure rate of 15.6% (28/180) in this retrospective cohort was similar to the failure rate 16.7% (4/24) in the registry cohort. The 3-year OS, DFS, CFS and LFR in the respective cohort were 90%, 80%, 88% and 15% respectively (See **Figure 1** for DFS and LFR). Compared to the registry cohort, no difference in OS, DFS, CFS and LFR was observed (See **Figures 1A, B** for DFS and LF).

**Table 4:** Comparison of patient and treatment characteristics between registry participants (n = 24) and patients in retrospective series (n = 180).

Patient/treatment characteristic	p-value*
Patient characteristic	
Age (years)	0.4
Sex (Female *vs.* Male)	0.3
ECOG (0-1, 2, 3)	0.5
Site (categories)	0.9
HIV positive (Yes *vs.* No)	0.6
T category (T1-4)	0.9
N category (N0 *vs.* N1)	0.9
M-Stage (categories 0-1)	0.4
Size of primary (cm)	0.6
Size of largest lymph node (cm)	0.8
Diversion preCRT (Yes *vs.* No)	0.5
Treatment Characteristic	
RT planned dose (Gy)	0.06
Fractions planned	0.1
RT given dose (Gy)	0.1
Fractions given	0.2
Concurrent chemotherapy (Yes *vs.* No)	0.1

*Two-sided p-value < 0.05% is considered statistically significant, Wilcoxon rank-sum test or Fisher exact test for continuous or categorical variables.

Upon multivariate analysis, increasing age (p = 0.007) and N category (N+ *vs.* N0, p = 0.02) were significant for worse OS in the retrospective cohort. Increasing size of the primary was the only significant factor for worse DFS (p <0.0001) and LFR (p = 0.0017). Since tumor size and N category were independent variables for worse outcome, we matched 48 patients from the retrospective cohort using tumor size and N category and compared the outcome to the 24 registry participants. **Table 5** outlines the tumor size and N category for these patients. The additional patient and tumor characteristics of the matched pairs are presented in **Supplementary Table 1**. The 3-year OS, DFS, CFS and LFR of the 48 matched patients were 89%, 76%, 87% and 16% respectively. In this retrospective to prospective 2 to 1 match pair comparison, there was again no difference observed in OS, DFS, CFS and LFR (See **Figures 1C, D** for DFS and LFR).

**Table 5:** Tumor size and N-category matching factors for prospective registry and retrospective patients.

	Registry (n = 24)	Retrospective (n = 48)	p-value*
Tumor size (cm)			0.4
< 2	1 (4.2%)	4 (8.3%)	
2 -< 4	9 (37.5%)	16 (33.3%)	
4 -<6	11 (45.8%)	15 (31.3%)	
≥ 6	3 (12.5%)	13 (27.1%)	
N-stage			0.6
0	12 (50.0%)	21 (43.8%)	
1-3	12 (50.0%)	27 (56.2%)	

n = number of patients.

*Two-sided p-value < 0.05% is considered statistically significant, Fisher exact test.

Given the retrospective grading of acute toxicities in the historic series of patients, only grade ≥3 acute toxicities were assessed. A comparison of grade ≥3 acute toxicities between the registry and retrospective patients is outlined in **Supplementary Table 2**. Specifically, with respect to acute hematologic toxicities, 34 (19%) had grade ≥3 neutropenia and 13 (14%) had grade ≥3 febrile neutropenia in the retrospective cohort. No grade 3 neutropenia or febrile neutropenia was observed in any of the registry participants (p = 0.02 and 0.05 respectively compared to the retrospective patients).

## Discussion

Historically, anal cancer at our institution was managed by split course CRT with large parallel opposed fields to cover the primary and regional nodes. Since IMRT became standard in 2011 for anal cancer at our cancer centre, patients had been managed by a prospectively designed protocol of CRT with total RT doses escalated according to increasing tumor stages. With the migration to IMRT, RT was planned without a gap. Unfortunately, 19% of patients developed grade ≥3 neutropenia and 14% had grade ≥3 febrile neutropenia despite IMRT. Similar acute hematological toxicities were reported in other IMRT studies (17, 18).

The incidence of febrile neutropenia was generally low in anal cancer patients where there was a cap in the chemotherapy drug doses (10). In the UK ACTII trial where the concurrent chemotherapy consisted of MMC (12 mg/m^2^ with a maximum dose of 20 mg) on day 1 only, and was omitted on week 5, the incidence of febrile neutropenia <1% (1 of 226) (9).

Given the emerging evidence that HPV-associated cancers including anal cancers are associated with increased radiosensitivity and improved prognosis (12, 13, 17) we instituted a prospective anal cancer registry study where the CRT protocol was tailored according to tumor stage and HPV status. The goal was to de-escalate the RT doses to determine if toxicities could be reduced without negative influence on tumor control in patients with HPV+ anal cancer. Further, to reduce the incidence of hematological toxicities and febrile neutropenia, the concurrent chemotherapy consisted of a single dose of MMC tailored after the UK ACTII trial (9). To the best of our knowledge, this represents the first analysis of dose de-escalation based on HPV status. A number of dose de-escalation studies are currently in progress for anal cancer such as the DECREASE trial (NCT04166318) and the ACT4 trial. It should be noted these studies are limited to patients with early (T1-2 N0) anal cancer irrespective of HPV status (18).

Despite de-escalation in RT dose and concurrent chemotherapy for the HPV+ patients, cancer outcome in the prospective registry patients appears remarkably similar when compared with the retrospective series of patients, and in the 2 to 1 match comparison. In the retrospective patients, 24% had a treatment gap >5 days due to acute toxicities. In cervical cancer, prospective data from EMBRACE studies have demonstrated impact of overall treatment time on survival, and delay in treatment appears to be detrimental (19). There are no prospective studies showing such effect of overall treatment time in anal cancer patients. It remains possible however that de-escalation of CRT reduces the potential of treatment prolongation and improves outcome. No grade ≥3 neutropenia and febrile neutropenia was observed in the registry participants. Among the 20 HPV+ patients, only 2 local failures were observed despite RT dose de-escalation and using a single dose of MMC. Two of 4 HPV− patients failed locally despite higher RT doses. A definite conclusion cannot be drawn however given the very small number of HPV− patients.

Although patient and tumor characteristics of the retrospective patients were similar to the registry patients, we acknowledge that there are likely to be some unknown selection biases and differences. In the retrospective cohort, only 25 patients had p16 immunohistochemistry performed, and 22 demonstrated immunoreactivity. Only 9 had HPV typing performed and they all were HPV+. It is thus likely that both cohorts had similar proportions of HPV+ and HPV− cancers. Although the staging distribution in the cohorts appeared similar, the retrospective cohort of patients were staged using CT and MRI only but not PET-CT. For N category, we reassigned them using the current TNM staging system as N0, N1a, N1b or N1c as they had been previously staged as N0, N1, N2 or N3. All toxicity grades were documented prospectively in the registry patients. We thus only analyzed grade ≥3 toxicities given the retrospective nature of the review of toxicities in this cohort of patients.

In the prospective registry patients, we did not observe any local failures beyond one year, whereas in the retrospective patients, local failure rates appeared to plateau off at 3 years. This may be due to intensive restaging using MRI and CT post CRT in the registry participants. This could also be an artifact given the small number of participants in the registry study, and it remains possible that additional local failures can be observed with longer follow-up.

The majority of anal cancers (62-97%) demonstrate immunoreactivity for p16 (13). Immunoreactivity of p16 has demonstrated to be a predictor of improved outcome after CRT in many studies. In a recent meta-analysis, patients with HPV+/p16+ anal cancer had the best OS and DFS whereas the prognosis in HPV+/p16− and HPV−/p16+ patients was not as favorable (13). Immunochemistry for p16 without HPV status may thus not be sufficient to predict prognosis. It is of interest that there was 100% concordance of p16 immunoreactivity with HPV status on PCR in our registry participants. Until further data are available, it may be premature to substitute p16 immunochemistry for HPV typing using PCR.

At our institution, cancer patients are encouraged to complete the Edmonton Symptom Assessment System (ESAS) at initial consultation, weekly during radiation, and at subsequent follow-up visits to assess symptom burden. ESAS however was not mandatory in the registry protocol. In the future, anal cancer specific Quality of Life Group questionnaire such as the ESAS or EORTC QLQ-ANL27 (20) could be used for patients for symptom and quality of life assessment before, during and after CRT to determine if de-escalation CRT protocols reduce the negative impact of on toxicities and quality of life in HPV+ patients.

In conclusion, dose de-escalation of CRT for HPV+ anal cancer may result in decreased acute toxicities and similar oncologic outcomes compared to standard CRT protocols. Based on these results, we will continue to accrue patients to the prospective registry without modification of the CRT protocol.

## Data availability statement

The original contributions presented in the study are included in the article/**Supplementary Material**. Further inquiries can be directed to the corresponding author.

## Ethics statement

The studies involving human participants were reviewed and approved by Sunnybrook Research Ethics Board. The patients/participants provided their written informed consent to participate in this study.

## Author contributions

WC, AT, YU, KC, CE, AK, MP, JP, and CW were responsible for the conceptualization, methodology, investigation and analysis of the research. JW was responsible for the pathology component and LZ performed the statistical analysis of the study. All authors participated in the writing, review and editing of the manuscript. CW was responsible for the overall management and coordination of the research. All authors approved the submitted version.
