# Supplementary material for: Risk-adjusted chemoradiation according to human papilloma viral status for anal cancer: a pilot study
Source: Front Oncol. 2023 Jun 29;13:1183854. doi: 10.3389/fonc.2023.1183854 (PMC10346840; doi:10.3389/fonc.2023.1183854)
Supplement: Supplementary file 1 [file DataSheet_1.docx]

**Supplementary Table 1.** Patient and tumor characteristics of retrospective cohort (n = 180).

| Sex |  |  |
| --- | --- | --- |
| Male | 52 | (29%) |
| Female | 128 | (71%) |
| ECOG |  |  |
| 0-1 | 159 | (88%) |
| 2 | 14 | (8%) |
| 3 | 2 | (1%) |
| Unknown | 5 | (3%) |
| P16+ |  |  |
| No | 3 | (2%) |
| Yes | 22 | (12%) |
| Unknown | 155 | (86%) |
| Site |  |  |
| Anal canal | 155 | (86%) |
| Perianal skin | 24 | (13%) |
| HIV positive |  |  |
| No | 168 | (93%) |
| Yes | 12 | (7%) |
| T-Stage |  |  |
| 1 | 18 | (10%) |
| 2 | 91 | (51%) |
| 3 | 38 | (21%) |
| 4 | 33 | (18%) |
| N-Stage |  |  |
| 0 | 91 | (51%) |
| 1a-c | 89 | (49%) |
| M-Stage |  |  |
| 0 | 177 | (98%) |
| 1* | 3 | (2%) |

*common iliac metastases

**Supplementary Table 2.** Comparison of patient and treatment characteristics between registry and 2 to 1 matched retrospective patients.

|  | Prospective  (n = 24) | Retrospective (n = 48) | p-value* |
| --- | --- | --- | --- |
| Age (years) |  |  | 0.9 |
| Mean ± SD | 67 ± 10 | 67 ± 13 |  |
| Median (Inter-quartiles) | 65 (61, 73) | 65 (56, 75) |  |
| Min, Max | 49, 86 | 38, 92 |  |
| Sex |  |  | 0.4 |
| Male | 4 (17%) | 14 (29%) |  |
| Female | 20 (83%) | 34 (71%) |  |
| ECOG |  |  |  |
| 0-1 | 21 (88%) | 43 (89%) | 0.9 |
| 2 | 2 (8%) | 3 (6%) |  |
| 3 | 1 (4%) | 2 (4%) |  |
| Site |  |  | 0.6 |
| Anal canal | 21 (88%) | 41 (85%) |  |
| Perianal skin | 3 (12%) | 7 (14%) |  |
| HIV positive |  |  | 0.7 |
| No | 23 (96%) | 44 (92%) |  |
| Yes | 1 (4%) | 4 (8%) |  |
| T-category |  |  | 0.7 |
| T1 | 1 (4%) | 6 (13%) |  |
| T2 | 14 (58%) | 23 (48%) |  |
| T3 | 5 (21%) | 9 (19%) |  |
| T4 | 4 (17%) | 10 (21%) |  |
| N-category |  |  | 0.2 |
| N0 | 12 (50%) | 21 (44%) |  |
| N1a | 5 (21%) | 16 (33%) |  |
| N1b | 2 (8%) | 0 (0%) |  |
| N1c | 5 (21%) | 11 (23%) |  |
| N-Stage combined categories |  |  | 0.7 |
| N0 | 12 (50%) | 21 (44%) |  |
| N1 | 12 (50%) | 27 (56%) |  |
| M-Stage |  |  | 1.0 |
| 0 | 23 (96%) | 46 (96%) |  |
| 1 | 1 (4%) | 2 (4%) |  |
| Size of primary (cm) |  |  | 0.9 |
| Mean ± SD | 4.5 ± 1.9 | 4.4 ± 2.1 |  |
| Median (Inter-quartiles) | 4.0 (3.3, 5.3) | 4.3 (3.0, 6.0) |  |
| Min, Max | 1.6, 9.1 | 1.0, 10.0 |  |
| Tumor size categories |  |  | 0.4 |
| < 2 cm | 1 (4%) | 4 (8%) |  |
| 2 - < 4 cm | 9 (38%) | 16 (33%) |  |
| 4 - < 6 cm | 11 (46%) | 15 (31%) |  |
| ≥ 6 cm | 3 (12%) | 13 (27%) |  |
| Size of largest node (cm) |  |  | 1.0 |
| n | 12 | 28 |  |
| Mean ± SD | 2.0 ± 1.5 | 1.7 ± 1.1 |  |
| Median (Inter-quartiles) | 1.5 (0.8, 3.0) | 1.4 (1.1, 1.9) |  |
| Min, Max | 0.6, 5.3 | 0.7, 5.6 |  |
| Diversion preCRT |  |  | 0.6 |
| No | 23 (96%) | 47 (98%) |  |
| Yes | 1 (4%) | 1 (2%) |  |
| ***Treatment Characteristics*** |  |  |  |
| RT planned dose (Gy) |  |  | 0.2 |
| n | 24 | 48 |  |
| Mean ± SD | 53.6 ± 3.7 | 56.5 ± 6.2 |  |
| Median (Inter-quartiles) | 55.8 (50.4, 55.8) | 54.0 (54.0, 63.0) |  |
| Min, Max | 45.0, 63.0 | 45.0, 63.0 |  |
| Fractions planned |  |  | 0.3 |
| n | 24 | 48 |  |
| Mean ± SD | 30 ± 2 | 31 ± 4 |  |
| Median (Inter-quartiles) | 31 (28, 31) | 30 (30, 35) |  |
| Min, Max | 25.0, 35.0 | 20.0, 35.0 |  |
| RT given dose (Gy) |  |  | 0.3 |
| n | 24 | 48 |  |
| Mean ± SD | 53.6 ± 3.7 | 55.9 ± 6.8 |  |
| Median (Inter-quartiles) | 55.8 (50.4, 55.8) | 54.0 (54.0, 63.0) |  |
| Min, Max | 45.0, 63.0 | 41.4, 64.8 |  |
| Fractions given |  |  | 0.3 |
| n | 24 | 48 |  |
| Mean ± SD | 29.75 ± 2.05 | 30.73 ± 4.35 |  |
| Median (Inter-quartiles) | 31.0 (28.0, 31.0) | 30.0 (30.0, 35.0) |  |
| Min, Max | 25.0, 35.0 | 20.0, 36.0 |  |
| Overall treatment time (days) |  |  | 0.003 |
| n | 24 | 48 |  |
| Mean ± SD | 42.2 ± 5.0 | 50.4 ± 12.7 |  |
| Median (Inter-quartiles) | 43.0 (39.5, 45.0) | 50.0 (43.0, 53.5) |  |
| Min, Max | 28.0, 52.0 | 32.0, 87.0 |  |
| Treatment gap >5 days |  |  | 0.01 |
| No | 23 (96%) | 34 (71%) |  |
| Yes | 1 (4%) | 14 (29%) |  |
| Treatment gap (> 5 days) |  |  | 0.4 |
| n | 1 | 14 |  |
| Mean (days) ± SD | 9 ± NA | 17.3 ± 7.6 |  |
| Median (Inter-quartiles) | 9 (9, 9) | 17 (13, 21) |  |
| Min, Max | 9, 9 | 6, 33 |  |
| Concurrent chemotherapy |  |  | 0.2 |
| No | 4 (17%) | 3 (6%) |  |
| Yes | 20 (83%) | 45 (94%) |  |

***** Two-sided p-value < 0.05% is considered statistically significant, Wilcoxon rank-sum test or Fisher exact test for continuous or categorical variables

**Supplementary Table 3.** Comparison of grade ≥3 acute toxicities between prospective registry and retrospective patients

| Toxicity grades ≥3 | Registry (n = 24) | Retrospective (n = 180) | p-value* |
| --- | --- | --- | --- |
| Vomiting |  |  | 0.4 |
| No | 24 (100%) | 175 (97%) |  |
| Yes | 0 (0%) | 5 (3%) |  |
| Diarrhea |  |  | 0.1 |
| No | 24 (100%) | 159 (88%) |  |
| Yes | 0 (0%) | 21 (12%) |  |
| Stomatitis |  |  | 0.9 |
| No | 23 (96%) | 171 (95%) |  |
| Yes | 1 (4%) | 9 (5%) |  |
| Proctitis |  |  | 0.7 |
| No | 24 (100%) | 179 (99%) |  |
| Yes | 0 (0%) | 1 (1%) |  |
| Cystitis |  |  | NA |
| No | 24 (100%) | 180 (100%) |  |
| Yes | 0 (0%) | 0 (0%) |  |
| Pain |  |  | 0.4 |
| No | 21 (88%) | 143 (79%) |  |
| Yes | 3 (12%) | 37 (21%) |  |
| Skin |  |  | 0.1 |
| No | 17 (71%) | 152 (84%) |  |
| Yes | 7 (29%) | 28 (16%) |  |
| Anemia |  |  | 0.8 |
| No | 22 (92%) | 162 (90%) |  |
| Yes | 2 (8%) | 18 (10%) |  |
| Neutropenia |  |  | 0.02 |
| No | 24 (100%) | 146 (81%) |  |
| Yes | 0 (0%) | 34 (19%) |  |
| Thrombocytopenia |  |  | 0.2 |
| No | 24 (100%) | 165 (92%) |  |
| Yes | 0 (0%) | 15 (8%) |  |
| Febrile Neutropenia |  |  | 0.05 |
| No | 24 (100%) | 155 (86%) |  |
| Yes | 0 (0%) | 25 (14%) |  |
| Any grades ≥3 toxicity |  |  | 0.5 |
| No | 16 (67%) | 103 (57%) |  |
| Yes | 8 (33%) | 77 (43%) |  |

*Two-sided p-value < 0.05% is considered statistically significant, Fisher exact test.
